# SARS-CoV-2 Variants in Paraguay: Detection and Surveillance with an Economical and Scalable Molecular Protocol

**DOI:** 10.3390/v14050873

**Published:** 2022-04-22

**Authors:** Magaly Martinez, Phuong-Vi Nguyen, Maxwell Su, Fátima Cardozo, Adriana Valenzuela, Laura Franco, María Eugenia Galeano, Leticia Elizabeth Rojas, Chyntia Carolina Díaz Acosta, Jonás Fernández, Joel Ortiz, Florencia del Puerto, Laura Mendoza, Eva Nara, Alejandra Rojas, Jesse J. Waggoner

**Affiliations:** 1 Departamento de Biología Molecular y Biotecnología, Instituto de Investigaciones en Ciencias de la Salud, Universidad Nacional de Asunción, San Lorenzo 111241, Paraguay; imartinez@iics.una.py (M.M.); laurafpy@hotmail.com (L.F.); maruphd@hotmail.com (M.E.G.); letyroj@hotmail.com (L.E.R.); chyntiacarolinadiaz@gmail.com (C.C.D.A.); jonasmfb@gmail.com (J.F.); joelortizm15@gmail.com (J.O.); colepuerto@gmail.com (F.d.P.); megunara@hotmail.com (E.N.); 2 Department of Medicine, Division of Infectious Diseases, Emory University, 1760 Haygood Drive NE, Room E-169, Bay E-1, Atlanta, GA 30322, USA; phuong-vi.thi.nguyen@emory.edu (P.-V.N.); max.su@emory.edu (M.S.); 3 Departamento de Salud Pública, Instituto de Investigaciones en Ciencias de la Salud, Universidad Nacional de Asunción, San Lorenzo 111241, Paraguay; fati.cardozo@hotmail.com (F.C.); abvalenzuela80@gmail.com (A.V.); lauramendozatorres@gmail.com (L.M.); 4 Departamento de Producción, Instituto de Investigaciones en Ciencias de la Salud, Universidad Nacional de Asunción, Dr. Cecilio Báez y Dr. Villamayor, Campus Universitario, San Lorenzo 111241, Paraguay; 5 Department of Global Health, Rollins School of Public Health, Atlanta, GA 30322, USA

**Keywords:** SARS-CoV-2, variants, real-time RT-PCR, Paraguay

## Abstract

SARS-CoV-2 variant detection relies on resource-intensive whole-genome sequencing methods. We sought to develop a scalable protocol for variant detection and surveillance in Paraguay, pairing rRT-PCR for spike mutations with Nanopore sequencing. A total of 201 acute-phase nasopharyngeal samples were included. Samples were positive for the SARS-CoV-2 N2 target and tested with the Spike SNP assay to detect mutations associated with the following variants: alpha (501Y), beta/gamma (417variant/484K/501Y), delta (452R/478K), and lambda (452Q/490S). Spike SNP calls were confirmed using amplicon (Sanger) sequencing and whole-genome (Nanopore) sequencing on a subset of samples with confirmed variant lineages. Samples had a mean N2 Ct of 20.8 (SD 5.6); 198/201 samples (98.5%) tested positive in the Spike SNP assay. The most common genotype was 417variant/484K/501Y, detected in 102/198 samples (51.5%), which was consistent with the P.1 lineage (gamma variant) in Paraguay. No mutations (K417 only) were found in 64/198 (32.3%), and K417/484K was identified in 22/198 (11.1%), consistent with P.2 (zeta). Seven samples (3.5%) tested positive for 452R without 478K, and one sample with genotype K417/501Y was confirmed as B.1.1.7 (alpha). The results were confirmed using Sanger sequencing in 181/181 samples, and variant calls were consistent with Nanopore sequencing in 29/29 samples. The Spike SNP assay could improve population-level surveillance for mutations associated with SARS-CoV-2 variants and inform the judicious use of sequencing resources.

## 1. Introduction

**Figure 1:**
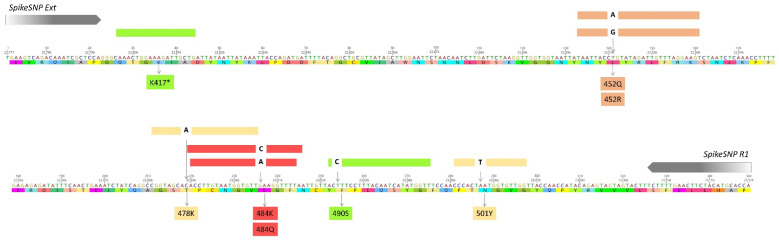
Graphical display of the 348-base-pair Spike SNP target, including the nucleic acid sequence and amino acid translation of the Wuhan-Hu-1 strain (NC_045512.2). The locations of the forward and reverse primer sequences and all hydrolysis probes are shown. Probe colors correspond to the fluorophores on each probe and the detection channel in the Rotor-Gene Q instrument. Detected nucleotide changes are displayed within the probe, and amino acid mutations are shown below the sequence. The probe for K417 is marked (*), as this is the only probe for which variant sequences result in signal loss and this has only been successfully designed with locked nucleic acid (LNA) bases.

Amidst the global pandemic caused by severe acute respiratory syndrome coronavirus 2 (SARS-CoV-2), numerous variants have emerged due to mutations in the positive-sense RNA genome. Variants of concern (VOCs) and variants of interest (VOIs) bear mutations that impact detection, treatment, clinical severity, transmission, and/or immune protection from prior infection or vaccination [1]. Whole-genome sequencing is the reference method for detecting and tracking variants, but this is a time- and resource-intensive process with the capacity varying markedly between regions [2,3,4,5]. It was estimated that 5% of all samples need to be sequenced to reliably detect variants at a prevalence of 0.1–1% [3], yet for many regions, this goal remains unattainable. Limited available sequencing capacity and overwhelming case numbers have motivated the development of real-time reverse transcription PCRs (rRT-PCRs) for the detection and surveillance of specific mutations found among VOCs/VOIs [6,7,8,9,10,11,12,13,14,15,16,17,18,19,20]. For these reasons, our group designed the Spike SNP assay, which is a multiplex rRT-PCR designed to detect specific mutations in the receptor binding motif of the Spike receptor binding domain (RBD; Figure 1) [20].

In Paraguay, the first confirmed SARS-CoV-2 infection was reported on 7 March 2020 [21], but 18 months into the pandemic, <0.1% of samples from the country could be sequenced and entered in the GISAID database (www.gisaid.org, accessed 1 March 2022) [22]. Published reports on case series in Paraguay have been limited to descriptive analyses of symptoms and disease outcomes [23,24]. As such, little is known about variants that circulated in the country during the beginning of the pandemic in 2020. However, data from 2021 show that variants originally detected in Brazil, such as the P.1 (gamma variant) and P.2 (zeta) lineages, circulated widely until the middle of that year (GISAID database). Nanopore sequencing on the portable MinION sequencer from ONT (Oxford Nanopore Technologies, Oxford, UK) became available in Asunción, the capital of Paraguay, to detect and monitor SARS-CoV-2 variants for the first time, where this technology provides lower-cost and more-rapid whole-genome sequencing compared to other methods [2,25]. The objective of the current study was to develop an economical and scalable protocol based on the Spike SNP assay and Nanopore sequencing to identify and monitor VOCs/VOIs in acute-phase samples in Paraguay.

## 2. Methods

### 2.1. Clinical Samples

Nasopharyngeal (NP) samples were obtained from residual baseline surveillance samples at the Instituto de Investigaciones en Ciencias de la Salud, Universidad Nacional de Asunción (IICS-UNA) Laboratory. Samples were de-identified, aliquoted, and stored at −80 °C until nucleic acid extraction. A convenience set of 201 SARS-CoV-2 positive samples from November 2020 to April 2021 was chosen for this study. Samples tested positive for the envelope gene target of the Charité protocol [26] with a cycle threshold (Ct) < 34 and were collected from different cities in Paraguay from symptomatic (*n* = 187) and asymptomatic (*n* = 14) individuals. This study was reviewed and approved by the IICS Scientific and Ethics Committee (P38/2020) and the Emory Institutional Review Board (study 00110736). Patient consent was waived under approval of IICS Scientific and Ethics Committee (P38/2020) due to all samples being de-identified remaining of diagnostics samples.

### 2.2. Spike SNP Design and Performance

Hydrolysis probes were designed to detect mutations associated with emerging variants of concern: L452Q (CAG > CTG) and F490S (UUU > UCU, C.37, lambda), E484Q (GAA > CAA, B.1.617.1, kappa), T478K (ACA > AAA, B.1.617.2, delta), and a modified T478K containing one mixed base in codon 477 for the detection of omicron. All new probes were designed as described [20], without locked nucleic acids (LNAs). Unmodified probes for 484K and 501Y were also designed and evaluated. New probes were individually evaluated using SARS-CoV-2 genomic RNA in singleplex assays. Probes that provided the most sensitive signal based on the cycle threshold (Ct) value, with preserved specificity, were selected. Subsequently, probes were tested in combination and compared side-by-side with the corresponding singleplex assays. Probe evaluations were performed by utilizing variant control samples that were kindly provided by the National Institutes of Health Variant Task Force and contained the following SARS-CoV-2 variants: alpha, beta, gamma, kappa, lambda, delta, and omicron. Variant control samples were heat-inactivated clinical samples that were prepared in the same manner as all clinical samples and then tested across a range of N2 Ct values from 20–36. Variants and lineages had been confirmed via sequencing (Helix, San Mateo, CA, USA).

Nucleic acids were extracted from all samples on an EMAG instrument (bioMérieux, Durham, NC) from 200 µL of NP swab and eluted in 50 µL. All rRT-PCRs were performed on a Rotor-Gene Q instrument (Qiagen, Germantown, MD, USA) using 20 µL reactions of the Luna Probe One-Step RT-qPCR Kit (New England Biolabs, Ipswich, MA, USA) and 5 µL of nucleic acid eluate. Each sample was tested in a duplex reaction for the SARS-CoV-2 nucleocapsid 2 target and RNase P (N2RP), as well as two different reactions of the Spike SNP assay, including probes for (1) K417, 452R, 484K, and 501Y, as described [20]; and (2) 452Q, 490S, and 484Q. During the study, two probes for 478K were designed, and all samples with 452R were tested in singleplex reactions with the original probe to search for the delta variant. Analytical performance of the second Spike SNP reaction was also evaluated following the inclusion of this probe. As sample collection pre-dated the emergence of omicron, samples were not re-tested with the revised 478K probe that was designed to also detect the mutation in this variant. The N2RP assay was performed as described [20,27]. For full Spike SNP methods, please see the Supplementary Materials. This study was performed in accordance with the STARD 2015 reporting guidelines (accessed 16 January 2022).

### 2.3. Sequencing

All rRT-PCR products from the first Spike SNP reaction (348-base-pair amplicon) were stored at 4 °C and later shipped to GeneWiz (South Plainfield, NJ, USA) for Sanger sequencing. This was performed to confirm the Spike SNP calls in the current study. The amplicon sequence was considered to be high quality and included for analysis if it achieved a quality score ≥ 40 and had base calls for all probe targets in at least one direction. A quality score ≥ 40, as defined by the company, signifies a mean probability of error ≤ 0.01% for each base call across the amplicon.

A representative subset of 29 samples underwent complete genome sequencing to confirm the variant lineage and identify mutations outside of the Spike SNP amplicon. Samples were selected to include the ancestral strain; alpha, gamma, and zeta variants; and one sample with the L452R mutation. Complete genome sequencing was performed following the Artic Network protocol using the V3 primer scheme [28]. The genomic libraries were prepared and then loaded on an R9.4.1 flow cell (ONT FLO-MIN106) for sequencing with a MinION device (MIN-101B, ONT, Oxford, UK). Basecalling, demultiplexing, and trimming were carried out using the Guppy toolkit integrated into the MinKNOW v.4.3.4 Nanopore software ( ONT, Oxford, UK). Genome assembly and consensus sequences were obtained with Nanopore EPI2ME v.3.4.1(ONT, Oxford, UK) (using Flye and Medaka respectively). The Nextclade tool was used for clade definition and mutation calling [29] and lineages were assigned using the Pangolin web server [30]. Sequences were deposited in the GISAID database.

### 2.4. Statistics

Basic statistical analyses were performed using GraphPad Prism version 9.0.2 (GraphPad Software, San Diego, CA, USA).

## 3. Results

### 3.1. Spike SNP Optimization

**Figure 2:**
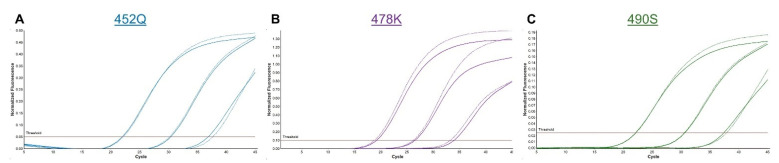
Addition of the 478K probe did not impact the signal from 452Q or 490S probes. Amplification curves are shown for 100-fold dilutions of C.37 (lambda) and B.1.617.2 (delta) samples tested together in a single run. The results were similar for (**A**) 452Q and (**C**) 490S mutations in C.37 with the probe for 478K (solid curves) or without (dotted curves). Results for B.1.617.2 were similar with the 478K probe (**B**) used in singleplex (dotted curves) or triplex reactions (solid curves). Nonspecific signals were not detected in any channel.

For the initial testing, a Spike SNP assay was developed that contained probes for 452Q, 484Q, and 490S. With the global emergence of B.1.617.2 and the lack of 484Q detection, the 478K probe was incorporated into the assay in place of 484Q. The addition of the original or revised 478K probe had no impact on 452Q or 490S detection (Figure 2), and despite overlapping sequences, the 478K and 484Q probes demonstrated no interference when combined in the same reaction (Figure S1). New probes for 484K and 501Y without LNA bases provided sensitive detection at both sites, though Ct values with the unmodified 484K probe were approximately two cycles later than those generated with the LNA probe (Figure S2). Testing of clinical samples in the current study was performed with the original 484K and 501Y probes to maintain consistency with existing protocols. All primers and probes used in or developed for this study are shown in Table 1, and their respective locations across the amplicon are displayed in Figure 1.

### 3.2. SNP Detection in Clinical Samples

**Figure 3:**
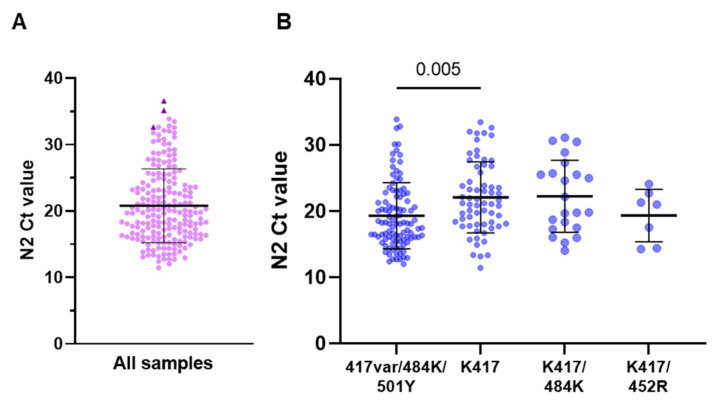
Distribution of N2 Ct values for (**A**) all samples and (**B**) the four most common genotypes detected. (**A**) N2 Ct values for all tested samples are displayed. Three samples that tested negative in the Spike SNP assay are highlighted (dark triangles) and had N2 Ct values of 32.6, 36.6, and 35.1. (**B**) N2 Ct values for the four most common genotypes detected. Bars represent mean Ct and standard deviation, and *p*-values for tested comparisons are displayed above the graph.

Clinical samples were collected between November 2020 and April 2021. A total of 113 participants were female (56.8% of the 199 with gender data available), and the age and day of symptoms at sample collection are shown in Table 2, along with the N2RP and Spike SNP assay results. The distribution of N2 target Ct values is shown in Figure 3 (Ct values provided in Table S1). The most common genotype identified was 417variant/484K/501Y (102/198 samples, 51.5%), which was consistent with B.1.351 (beta) and P.1 (gamma). Large numbers of cases were also detected with a signal for K417 only (64/198, 32.3%), which was consistent with a non-variant/ancestral lineage, and K417/484K (22/198, 11.1%), which was observed in P.2 (zeta), among others. Seven samples (3.5%) had the genotype K417/452R but tested negative with the 478K probe and were thus not consistent with B.1.617.2.

Samples with the 417variant/484K/501Y genotype had significantly lower N2 Ct values (mean 19.3, standard deviation 5.0) than all other samples (22.0, 5.4; *p* < 0.001; data not shown) and samples with K417 only (22.1, 5.4; *p* = 0.002; Figure 3B). No differences in patient age or day of symptoms were observed based on the genotype (*p* > 0.05 for all comparisons).

### 3.3. Amplicon and Whole-Genome Sequencing

**Figure 4:**
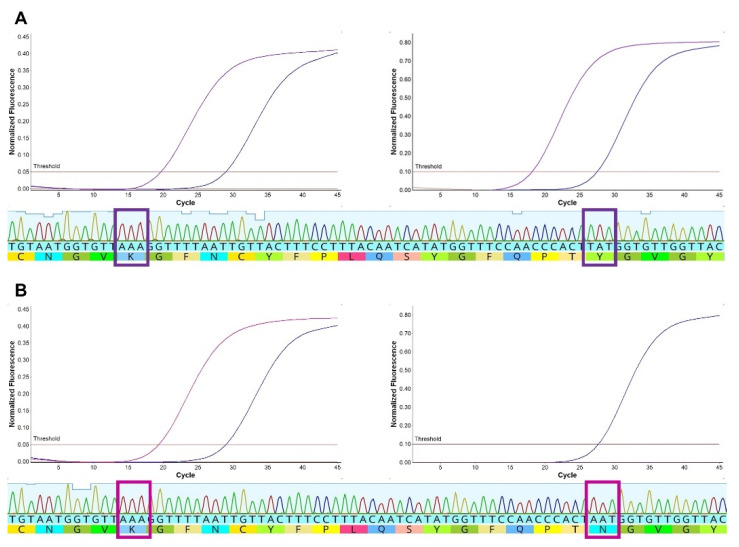
Spike SNP and amplicon sequencing results were concordant. Amplification curves and amplicon sequence tracings are shown for samples with mutations encoding (**A**) 484K and 501Y (purple curves) or (**B**) 484K only with N501 (cranberry). A beta variant control sample is shown for reference (blue curves). Bold squares highlight the SNP mutations in the amplicon sequence.

High-quality amplicon sequences were obtained from 181 samples (181/201, 90.0%) using Sanger sequencing, and the results confirmed Spike SNP genotype calls for all samples (Table 2). Characteristic findings for samples with 484K, with and without 501Y, are shown (Figure 4). All samples with variant calls at position 417 had the mutation AAG > ACG conferring K417T were observed in P.1. One sample had 484K detected in the Spike SNP assay (genotype K417/484K) but E484 was detected based on the consensus Sanger sequence. On review of the tracing, there was a mixture of G and A bases at the first position of codon 484, which is consistent with the evidence of a mixed viral population in this sample. Notably, this sample had a late Ct value for 484K (38.4) relative to K417 (34.3) when compared to other samples with this genotype (mean Ct difference 0.4, standard deviation 0.2). In addition to mutations targeted in the Spike SNP assay, four samples (2.2%) had a mutation conferring T478R (ACA > AGA) and one sample (0.5%) had a mutation resulting in N501T (AAU > ACU).

Whole-genome sequences for 29 samples were obtained using the Artic protocol. Ten different lineages were identified (Table 3) [30]. Nanopore sequencing confirmed Spike SNP genotype calls for all samples. Samples bearing genotype 417variant/484K/501Y belonged to the P.1 lineage and P.1.2 sub-lineage. The genotype of only K417 was found in samples of lineages B.1.1.28, B.1.1.33, B.1.1.277, and N3, all within the 20B Nextstrain clade and lineage B.1.499 (clade 20C) [29]. The P.2 lineage that also belongs to the 20B clade showed the K417/484K genotype. One sample with genotype K417/501Y was classified as B.1.1.7 (alpha). A lineage from 2020 that was still circulating during 2021, namely, A.2.5.2, was found in one sample with genotype K417/452R.

## 4. Discussion

Among individuals with a SARS-CoV-2 infection in Paraguay, the Spike SNP assay provided accurate detection of targeted mutations in the RBD and, in conjunction with the employed sequencing protocol, confirmed the predominance of B.1.1.28, P.2, and P.1 in this population between November 2020 and April 2021, as well as the relative absence of B.1.1.7. Given the frequent travel between Paraguay and Brazil, the B.1.1.28 lineage was expected to account for a high proportion of cases in late 2020 and early 2021, to be later replaced by P.1 and P.2. Samples with P.1 had lower Ct values in our population. A similar finding was initially documented in Brazil and likely resulted in increased transmissibility of the gamma variant with the replacement of other lineages [31,32]. The P.1 and P.2 lineages were readily differentiated from one another in the Spike SNP assay, and in a real-world scenario, the N2RP and Spike SNP assays could be performed sequentially to diagnose SARS-CoV-2 infection, identify a set of common mutations, and select samples for downstream sequencing [4,5,20].

For the current study, new probes were generated for mutations that occurred within the target region and are associated with emerging VOCs/VOIs, including delta, lambda, and omicron variants (Figure 1). As new designs do not involve separate primer–probe sets, the assay can be readily modified and reconfigured to include probes that match circulating variants in a particular region. All new probes were designed without LNA bases yet demonstrated similar performances to previous designs (Figure S1). Although LNA bases were shown to be important for the detection of K417 mutations, avoiding their use in other probes improves access to and reduces costs for these oligos. An additional benefit to the current design is that up to four mutations are targeted in a single reaction, which has been found to improve variant classification [9]. Other published rRT-PCR protocols for the detection of a similar number of mutations generate two or more amplicons in single-reaction [12,15,17] or multiple-reaction designs [6,8,14,16], and such protocols may be more difficult to modify in response to the emergence of new variants.

As shown in the current study, the Spike SNP assay can be paired with whole-genome sequencing on a MinION device to provide an economical and tractable molecular protocol for SARS-CoV-2 variant detection. Nanopore sequencing has become a common method worldwide and particularly in locations where the use of short-read sequencing platforms is less feasible. MinION sequencing was well described for SARS-CoV-2 and provides formal confirmation of detected variants and lineages, which cannot be achieved using rRT-PCR alone [4,5,25,31]. However, the Spike SNP assay may provide more sensitive detection of specific mutations than can be achieved with current sequencing protocols. Amplicon sequencing was performed in the current study to confirm Spike SNP calls for assay validation. While this provides less information than whole-genome sequencing, it serves to differentiate B.1.351 from P.1 and identify additional mutations in the receptor-binding motif. In our study, four individuals had the T478R mutation; the whole genome of two of them was sequenced and assigned to the B.1.499 lineage. This lineage circulated widely in Argentina during 2020, but the mutation T478R was not reported. While this mutation is distinct from T478K found in the B.1.617.2 (delta) and B.1.1.529 (omicron), mutations at position 478 have developed in vitro and confer decreased antibody neutralization, indicating that such mutations may benefit the virus through immunity escape [33,34]. Finally, one individual was identified with a possible mixed infection or a minor variant bearing 484K [35,36]. Such infections do not appear to cause more severe clinical disease [35], but this case highlights one potential benefit of rRT-PCR surveillance if key mutations can be detected that are not observed in the consensus sequence.

The degree of multiplexing possible in the Spike SNP assay is limited by the number of channels available in common real-time PCR instruments (4–5), as well as the defined 348-base-pair target region. Based on the results of testing for 478K and 484Q in a single reaction, overlapping probes did not impact the performance (Figure S1). However, multiplexed detection of key mutations outside of the Spike SNP target region would require additional primer–probe sets and assay optimization. A limitation of the current study was the utilization of retrospective convenience samples to evaluate the testing protocol. Although new data on SARS-CoV-2 variants in Paraguay were generated, we cannot calculate variant prevalence from these data.

In conclusion, the Spike SNP assay provided accurate detection of mutations that were associated with VOCs/VOIs in Paraguay. This can be implemented in SARS-CoV-2 testing protocols to triage samples for whole-genome sequencing in the MinION platform or direct amplicon sequencing, which provides additional information. The assay can be implemented in any laboratory performing rRT-PCR to improve the surveillance for these mutations and inform the judicious use of scarce sequencing resources.

## Figures and Tables

**Table 1:** Spike SNP assay primers and probes used in the current study.

Name	Sequence ^a^	Concentration (nM) ^b^	Location (5′–3′) ^c^
Primers			
SpikeSNP Ext ^d^	TGAAGTCAGACAAATCGCTCC	400	22,777–22,797
SpikeSNP R1 ^d^	TGGTGCATGTAGAAGTTCAAAAG	400	23,103–23,125
LNA Probes			
K417 ^d^	FAM-CAAACTGGA+A+**A**+**G**+ATTGCTG-3IABkFQ	200	22,802–22,820
484K ^d^	Cy5-ACCTTGTAATGGTGT+T+**A**+AAGGTTTT-3IAbRQSp	200	22,996–23,020
501Y ^d^	HEX-CCCAC+T+**T**+ATGGTGTTGG-3IABkFQ	200	23,057–23,073
Unmodified Probes			
452R ^d^	CFR610-ATAATTACC**G**GTATAGATTGTTTAGGAAGT-BHQ-2	200	22,908–22,937
484K	Q670-CACCTTGTAATGGTGTT**A**AAGGTTTTAA-BHQ-2	200	22,995–23,022
484Q	Q670-CACCTTGTAATGGTGTT**C**AAGGTTTTAA-BHQ-2	200	22,995–23,022
501Y	CFO560-CAACCCACT**T**ATGGTGTTGGTTAC-BHQ-1	200	23,054–23,077
452Q	CFR610-ATAATTACC**A**GTATAGATTGTTTAGGAAGT-BHQ-2	200	22,908–22,937
490S	FAM-ACT**C**TCCTTTACAATCATATGGTTTCC-BHQ-1	200	23,028–23,054
478K (original)	CFO560-CGGTAGCA**A**ACCTTGTAATGGTG-BHQ-1	200	22,987–23,009
478K (revised)	CFO560-CGGTARCA**A**ACCTTGTAATGGTG-BHQ-1	200	22,987–23,009

Abbreviations: BHQ, black hole quencher; CFO560, CAL Fluor Orange 560; CFR610, CAL Fluor Red 610; Cy5, Cyanine 5; FAM, Fluorescein; IABkFQ/IAbRQSp, Iowa Black quenchers; Q670, Quasar 670; ^a^ probe sequences listed 5′-fluorophore-sequence-quencher-3′; “+” before base indicates a locked nucleic acid, bold and underlined base indicates the targeted single nucleotide change; ^b^ concentration in the final reaction mixture; ^c^ location in Wuhan-Hu-1 complete genome sequence (NCBI Reference Sequence: NC_045512.2); ^d^ previously published, presented for clarity.

**Table 2:** Genotypes detected in the Spike SNP assay, patient demographics, and the percent confirmed using amplicon sequencing.

Category	n (%)	Age, Years, Mean (SD) ^a^	Day of Symptoms, Mean (SD) ^b^	Ct, Mean (SD) ^c^	Sequencing Confirmed, n/N (%) ^d^
N2 detected	201 (100)	42.6 (16.4)	4.8 (3.0)	20.8 (5.6)	─
Spike SNP detected	198 (98.5)	42.5 (16.4)	4.8 (3.0)	20.6 (5.3)	181/181 (100)
Genotype ^e^					
417var/484K/501Y	102 (51.5)	42.8 (14.9)	4.5 (2.4)	19.3 (5.0)	96/96 (100)
K417	64 (32.3)	42.6 (18.8)	5.1 (3.7)	22.1 (5.4)	56/56 (100)
K417/484K	22 (11.1)	37.4 (14.2)	4.8 (2.7)	22.2 (5.4)	20/20 (100) ^f^
K417/452R	7 (3.5)	54.0 (15.8)	5.0 (4.6)	19.4 (4.0)	7/7 (100)
K417/490S	1 (0.5)	41	4	15.2	1/1 (100)
K417/501Y	1 (0.5)	68	5	19.7	1/1 (100)
K417/484K/501Y	1 (0.5)	19	10	32.1	NA

Abbreviations: var, variant; SD, standard deviation; ^a^ age available for 194/201 (96.5%) samples; ^b^ day of symptoms available for 136/201 (67.7%) samples; ^c^ Ct value in the N2 assay; ^d^ number with sequence confirmed (n) over the number of amplicons with high-quality sequence and base calls at each SNP position (N); ^e^ displayed as % of Spike SNP positive samples; ^f^ one sample had a mixed base (A/G) at the position targeted with the 484K probe.

**Table 3:** Spike mutations and lineages identified using genome sequencing.

Sample Code	Spike SNP Genotype	Nanopore Sequencing			
		GISAID Accession No.	Nextstrain CladeWHO Label	Pango Lineage	Spike Deletions/Amino Acid Substitutions ^a^
PY21-100	417var/484K/501Y	EPI_ISL_4071892	20J (V3) gamma	P.1	L18F/T20N/P26S/D138Y/R190S/**K417T/E484K/N501Y**/D614G/H655Y/T1027I/V1176F
PY21-9	417var/484K/501Y	EPI_ISL_2444778	20J (V3) gamma	P.1	L18F//T20N/P26S/D138Y/R190S/**K417T/E484K/N501Y**/D614G/H655Y/T1027I/V1176F
PY21-57	417var/484K/501Y	EPI_ISL_4137476	20J (V3) gamma	P.1	L18F/T20N/P26S/D138Y/R190S/**K417T/E484K/N501Y**/D614G/H655Y/T1027I/V1176F
PY21-58	417var/484K/501Y	EPI_ISL_4071897	20J (V3) gamma	P.1	L18F/T20N/P26S7D138Y/R190S/**K417T/E484K/N501Y**/D614G/H655Y/T1027I/V1176F
PY21-84	417var/484K/501Y	EPI_ISL_4071898	20J (V3) gamma	P.1	L18F/T20N/P26S/D138Y/R190S/**K417T/E484K/N501Y**/D614G/H655Y/T1027I/V1176F
PY21-16	417var/484K/501Y	EPI_ISL_4071893	20J (V3) gamma	P.1	L18F/T20N/P26S/D138Y/R190S/**K417T/E484K/N501Y**/D614G/H655Y/T1027I/V1176F
PY21-11	417var/484K/501Y	EPI_ISL_4071900	20J (V3) gamma	P.1	L18F/T20N/P26S/D138Y/L189F/R190S/**K417T/E484K/N501Y**/D614G/H655Y/T1027I/V1176F
PY21-90	417var/484K/501Y	EPI_ISL_4071899	20J (V3) gamma	P.1.2	L18F/T20N/P26S/T95I/D138Y/R190S/**K417T/E484K/N501Y**/D614G/H655Y/Q675H/T1027I/V1176F/V1228L
PY21-150	K417	EPI_ISL_2234899	20B	B.1.1.33	D614G
PY21-153	K417	EPI_ISL_2234891	20B	B.1.1.33	D614G
PY21-140	K417	EPI_ISL_2234880	20B	B.1.1.28	D614G/V1176F
PY21-145	K417	EPI_ISL_4084605	20B	B.1.1.28	D614G/V1176F
PY21-146	K417	EPI_ISL_2234881	20B	B.1.1.28	D614G/V1176F
PY21-98	K417	EPI_ISL_2444788	20B	B.1.1.28	D614G/V1176F
PY21-152	K417	EPI_ISL_2234885	20B	B.1.1.28	T22I/D614G/V1176F
PY21-144	K417	EPI_ISL_4071901	20B	B.1.1.28	D614G/N1135Y/V1176F
PY21-43	K417	EPI_ISL_4071895	20B	B.1.1.28	A575S/D614G/V1176F
PY21-44	K417	EPI_ISL_4084604	20B	B.1.1.28	T572I/D614G/V1176F
PY21-47	K417	EPI_ISL_4071894	20B	B.1.1.28	A575S/D614G/V1176F
PY21-148	K417	EPI_ISL_2234894	20B	B.1.1.28	V6F/L18F/D614G/V1176F
PY21-147	K417	EPI_ISL_2234887	20B	B.1.1.28	Q14H/D614G/D1153A/V1176F
PY21-154	K417	EPI_ISL_2234893	20B	B.1.1.277	D614G/V1176F
PY21-52	K417/484K	EPI_ISL_2444780	20B (zeta)	P.2	**E484K**/D614G/V1176F
PY21-157	K417/484K	EPI_ISL_2234879	20B (zeta)	P.2	L5F/**E484K**/D614G/A771S/V1176F
PY21-143	K417	EPI_ISL_2234889	20B	N.3	T76I/D614G
PY21-141	K417	EPI_ISL_2234883	20C	B.1.499	T478R/D614G
PY21-149	K417	EPI_ISL_2234897	20C	B.1.499	T478R/D614G
PY21-102	K417/452R	EPI_ISL_4071896	19B	A.2.5.2	**L452R**/D614G
PY21-156	K417/501Y	EPI_ISL_4084606	20I (V1) alpha	B.1.1.7	DEL69-70/DEL144**N501Y**/A570D/D614G/P681H/T716I/S982A/D1118H

Abbreviations: var, variant; WHO, World Health Organization; ^a^ bold text indicates amino acid substitutions detected using the Spike SNP assay.

## Data Availability

All supporting data necessary for interpretation are provided herein. Complete genome sequences were deposited in GISAID and accession numbers are shown in Table 3. Amplicon sequence tracings are available upon request.

## Supplementary Materials

The following supporting information can be downloaded at: https://www.mdpi.com/article/10.3390/v14050873/s1, File S1: Spike SNP Performance; Table S1: Spike SNP and N2RP line data for 201 clinical samples tested from Paraguay; Figure S1: 478K and 484Q probes do not interfere despite overlapping sequences; Figure S2: Performance of unmodified hydrolysis probes compared to probes that include LNA bases.
