# Supplementary material for: SARS-CoV-2 Variants in Paraguay: Detection and Surveillance with an Economical and Scalable Molecular Protocol
Source: Viruses. 2022 Apr 22;14(5):873. doi: 10.3390/v14050873 (PMC9145602; doi:10.3390/v14050873)
Supplement: Supplementary file 1 [file viruses-14-00873-s001.zip › viruses-1643158-supplementary.pdf]

viruses

MDPI

Supplementary Materials

SARS-CoV-2 Variants in Paraguay: Detection and Surveillance with an Economical and Scalable Molecular Protocol

File S1. Spike SNP Performance.

Spike SNP assays were performed as separate runs on the same day using the following conditions: 52 °C for 15 min, 94 °C for 2 min, and 45 cycles of 94 °C for 15 s and 60 °C for 60 s. At 60 °C, a fluorescent signal was acquired in all channels. Thresholds for each channel were set during assay optimization and used for all subsequent analyses. During the analysis of the Spike SNP data, outlier removal was performed for signals that did not reach 10% (K417, 490S) or 20% (452R, 484K, 501Y, 452Q, 484Q, 478K) of the maximum fluorescence in a given channel. Samples were considered positive in the Spike SNP assay if an exponential curve was generated with one or more probes.

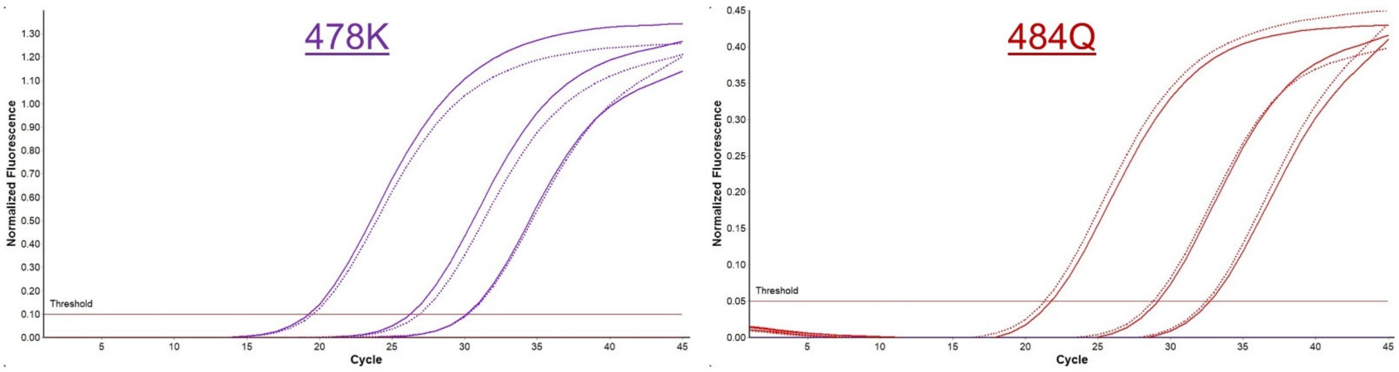

Figure S1. 478K and 484Q probes do not interfere with detection despite overlapping sequences. Dilutions of RNA from B.1.617.2 (delta variant, 478K) and B.1.617.1 (484Q) were tested in Spike SNP triplexes containing probes for 452Q, 490S, and either 478K or 484Q (solid curves), or a Spike SNP quadruplex assay containing all 4 probes on a single run (dotted curves). Amplification curves are shown for undiluted eluate, 100-fold and 1000-fold dilutions for each variant.

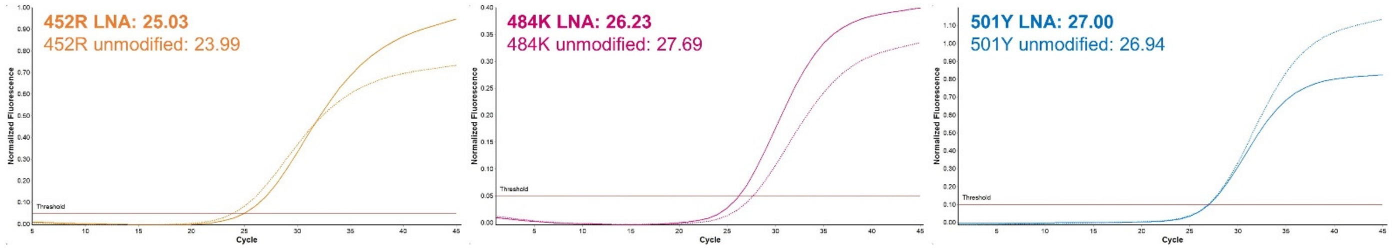

Figure S2. Performance of unmodified hydrolysis probes compared to probes that include LNA bases for the detection of mutations that cause 452R, 484K and 501Y. Controls containing the particular mutation were tested on a single run using both probes. Ct values are displayed. Curves generated from probes containing LNA bases are shown as solid lines; those generated with unmodified probes are displayed as thin, dotted lines.

Table S1. Spike SNP and N2RP line data for 201 clinical samples tested from Paraguay.

Código (PY21)	N2	RNase P	417K	452R	484K	501Y	452Q	484Q	490S	Qualitative Spike SNP Result	SNPs Confirmed (amplicon sequencing)
PY21-1	25.71	22.26	N	N	31.98	30.56	N	N	N	Pos	Yes
PY21-2	24.13	18.30	27.18	27.43	bleed	N	N	N	N	Pos	Yes
PY21-3	14.41	16.75	17.22	17.73	bleed	N	N	N	N	Pos	Yes
PY21-4	13.90	16.62	N	N	18.40	16.65	N	N	N	Pos	Yes
PY21-5	20.50	20.32	N	N	24.72	23.10	N	N	N	Pos	Yes
PY21-6	13.54	16.26	N	N	17.98	16.32	N	N	N	Pos	Yes
PY21-7	19.94	18.99	N	N	24.49	22.95	N	N	N	Pos	Yes
PY21-8	25.00	19.99	N	N	29.40	28.00	N	N	N	Pos	Yes
PY21-9	22.75	22.33	N	N	27.21	25.65	N	N	N	Pos	Yes
PY21-10	32.56	23.67	N	N	37.10	35.92	N	N	N	Pos	NA
PY21-11	20.42	21.01	N	N	24.90	23.45	N	N	N	Pos	Yes
PY21-12	26.71	21.44	N	N	32.04	30.68	N	N	N	Pos	Yes
PY21-13	21.39	20.69	N	N	25.15	23.63	N	N	N	Pos	Yes
PY21-14	24.25	20.83	N	N	28.27	26.86	N	N	N	Pos	Yes
PY21-15	29.22	17.77	N	N	34.78	33.56	N	N	N	Pos	NA
PY21-16	12.95	-	N	N	16.58	14.81	N	N	N	Pos	Yes
PY21-17	22.14	19.06	N	N	26.88	25.31	N	N	N	Pos	Yes
PY21-18	23.12	20.80	N	N	28.30	26.82	N	N	N	Pos	Yes
PY21-19	13.06	-	N	N	18.02	16.34	N	N	N	Pos	Yes
PY21-20	12.74	15.28	N	N	17.27	15.57	N	N	N	Pos	Yes
PY21-21	30.12	21.73	N	N	34.40	32.92	N	N	N	Pos	Poor Quality
PY21-22	20.01	20.34	N	N	24.61	23.05	N	N	N	Pos	Yes
PY21-23	12.76	15.60	N	N	17.26	15.51	N	N	N	Pos	Yes
PY21-24	18.07	20.77	N	N	21.91	20.35	N	N	N	Pos	Yes
PY21-25	18.73	16.36	22.35	N	22.94	N	N	N	N	Pos	Yes
PY21-26	18.34	21.58	21.14	N	21.82	N	N	N	N	Pos	Yes
PY21-27	23.89	21.75	26.13	N	N	N	N	N	N	Pos	Yes
PY21-28	18.02	17.09	20.67	N	N	N	N	N	N	Pos	Yes
PY21-29	13.18	15.56	15.76	N	N	N	N	N	N	Pos	Yes
PY21-30	19.75	18.55	22.06	N	N	N	N	N	N	Pos	Yes
PY21-31	32.03	18.46	36.66	N	N	N	N	N	N	Pos	NA
PY21-32	18.13	17.27	20.56	N	N	N	N	N	N	Pos	Yes
PY21-33	21.86	17.67	25.02	N	N	N	N	N	N	Pos	Yes
PY21-34	13.36	13.81	15.61	N	N	N	N	N	N	Pos	Yes
PY21-35	17.71	17.75	20.00	N	N	N	N	N	N	Pos	Yes
PY21-36	35.12	19.11	N	N	N	N	N	N	N	Neg	NA
PY21-37	20.10	18.38	23.06	N	N	N	N	N	N	Pos	Yes
PY21-38	14.05	14.23	16.31	N	16.93	N	N	N	N	Pos	Yes
PY21-39	18.85	18.56	21.70	N	N	N	N	N	N	Pos	Yes
PY21-40	11.45	15.20	13.46	N	N	N	N	N	N	Pos	Yes
PY21-41	15.23	15.29	17.94	N	N	N	N	N	20.08	Pos	Yes
PY21-42	28.35	20.53	31.24	N	N	N	N	N	N	Pos	Yes
PY21-43	20.88	18.21	23.65	N	N	N	N	N	N	Pos	Yes
PY21-44	21.07	19.85	23.54	N	N	N	N	N	N	Pos	Yes
PY21-45	33.46	22.47	37.97	N	N	N	N	N	N	Pos	NA
PY21-46	17.73	17.51	20.04	N	N	N	N	N	N	Pos	Yes

PY21-47	13.38	17.07	15.87	N	N	N	N	N	N	Pos	Yes
PY21-48	36.58	19.67	N	N	N	N	N	N	N	Neg	NA
PY21-49	24.60	20.98	27.53	N	27.90	N	N	N	N	Pos	Yes
PY21-50	26.73	20.10	30.04	N	N	N	N	N	N	Pos	Yes
PY21-51	28.67	23.28	N	N	33.11	31.81	N	N	N	Pos	Yes
PY21-52	19.80	17.30	22.62	N	23.03	N	N	N	N	Pos	Yes
PY21-53	22.69	16.63	25.79	N	N	N	N	N	N	Pos	Yes
PY21-54	22.81	21.79	N	N	26.81	25.37	N	N	N	Pos	Yes
PY21-55	18.52	18.25	N	N	21.64	20.17	N	N	N	Pos	Yes
PY21-56	24.98	20.71	28.28	N	28.56	N	N	N	N	Pos	Yes
PY21-57	15.78	15.30	N	N	18.85	17.23	N	N	N	Pos	Yes
PY21-58	16.97	16.73	N	N	20.77	19.33	N	N	N	Pos	Yes
PY21-59	30.11	17.75	N	N	35.32	34.64	N	N	N	Pos	Poor Quality
PY21-60	16.34	16.39	N	N	19.67	18.17	N	N	N	Pos	Yes
PY21-61	30.47	25.74	34.26	N	38.36	N	N	N	N	Pos	NA
PY21-62	27.65	17.16	31.02	N	N	N	N	N	N	Pos	Yes
PY21-63	25.50	20.98	28.72	N	29.03	N	N	N	N	Pos	Yes
PY21-64	27.76	17.83	30.96	N	N	N	N	N	N	Pos	Yes
PY21-65	32.12	19.33	37.27	N	35.75	35.53	N	N	N	Pos	NA
PY21-66	13.46	13.38	N	N	16.29	14.68	N	N	N	Pos	Yes
PY21-67	16.14	15.94	N	N	19.76	18.19	N	N	N	Pos	Yes
PY21-68	20.75	16.78	N	N	23.55	22.08	N	N	N	Pos	Yes
PY21-69	19.67	17.37	22.58	N	23.00	N	N	N	N	Pos	Yes
PY21-70	27.31	18.45	30.49	N	30.71	N	N	N	N	Pos	Yes
PY21-71	26.11	20.47	N	N	29.32	27.96	N	N	N	Pos	Yes
PY21-72	31.12	21.02	34.45	N	34.49	N	N	N	N	Pos	Yes
PY21-73	22.59	18.93	N	N	25.46	23.97	N	N	N	Pos	Yes
PY21-74	32.82	21.65	N	N	37.87	N	N	N	N	Pos	NA
PY21-75	16.68	16.84	N	N	19.84	18.34	N	N	N	Pos	Yes
PY21-76	16.73	16.80	N	N	20.71	19.29	N	N	N	Pos	Yes
PY21-77	16.29	17.07	N	N	19.49	17.90	N	N	N	Pos	Yes
PY21-78	13.83	14.75	N	N	16.82	15.16	N	N	N	Pos	Yes
PY21-79	18.64	17.90	N	N	21.30	19.80	N	N	N	Pos	Yes
PY21-80	27.00	19.87	30.24	N	N	N	N	N	N	Pos	Yes
PY21-81	12.02	16.81	N	N	14.96	13.28	N	N	N	Pos	Yes
PY21-82	14.39	14.80	N	N	18.25	16.65	N	N	N	Pos	Yes
PY21-83	18.24	17.68	N	N	21.63	20.13	N	N	N	Pos	Yes
PY21-84	18.14	16.15	N	N	21.19	19.65	N	N	N	Pos	Yes
PY21-85	15.90	15.25	N	N	19.24	17.61	N	N	N	Pos	Yes
PY21-86	15.26	15.42	N	N	18.43	16.79	N	N	N	Pos	Yes
PY21-87	18.17	17.16	N	N	21.36	19.80	N	N	N	Pos	Yes
PY21-88	23.18	22.09	N	N	26.34	24.92	N	N	N	Pos	Yes
PY21-89	27.49	19.10	N	N	31.19	29.78	N	N	N	Pos	Yes
PY21-90	15.33	20.54	N	N	19.29	17.75	N	N	N	Pos	Yes
PY21-91	18.09	16.31	N	N	21.68	20.13	N	N	N	Pos	Yes
PY21-92	17.78	17.18	21.04	N	N	N	N	N	N	Pos	Yes
PY21-93	16.73	17.34	19.09	N	N	N	N	N	N	Pos	Yes
PY21-94	19.15	16.68	21.90	N	N	N	N	N	N	Pos	Yes
PY21-95	23.17	19.33	26.31	N	N	N	N	N	N	Pos	Yes
PY21-96	23.61	19.09	26.66	N	N	N	N	N	N	Pos	Yes
PY21-97	25.70	19.58	28.84	N	29.24	N	N	N	N	Pos	Yes
PY21-98	17.41	16.99	20.49	N	N	N	N	N	N	Pos	Yes

PY21-99	29.19	18.62	32.61	N	N	N	N	N	N	Pos	NA
PY21-100	16.07	15.54	N	N	20.32	18.79	N	N	N	Pos	Yes
PY21-101	18.35	17.88	21.64	N	N	N	N	N	N	Pos	Yes
PY21-102	21.03	20.02	23.46	23.87	bleed	N	N	N	N	Pos	Yes
PY21-103	30.65	21.78	34.72	N	34.90	N	N	N	N	Pos	NA
PY21-104	15.42	15.59	18.57	N	N	N	N	N	N	Pos	Yes
PY21-105	22.81	20.08	25.49	N	N	N	N	N	N	Pos	Yes
PY21-106	28.74	27.92	32.90	N	N	N	N	N	N	Pos	Yes
PY21-107	23.27	19.81	27.93	N	N	N	N	N	N	Pos	Yes
PY21-108	32.62	29.92	35.23	N	N	N	N	N	N	Pos	Yes
PY21-109	14.92	14.57	17.28	N	N	N	N	N	N	Pos	Yes
PY21-110	31.47	16.53	34.51	N	N	N	N	N	N	Pos	NA
PY21-111	19.77	18.28	23.34	N	23.79	N	N	N	N	Pos	Yes
PY21-112	16.98	17.44	20.06	N	N	N	N	N	N	Pos	Yes
PY21-113	21.02	17.16	23.32	N	N	N	N	N	N	Pos	Yes
PY21-114	17.27	16.03	20.20	N	20.67	N	N	N	N	Pos	Yes
PY21-115	28.88	18.79	31.64	N	31.83	N	N	N	N	Pos	Yes
PY21-116	17.97	17.79	20.64	N	N	N	N	N	N	Pos	Yes
PY21-117	25.52	18.77	28.61	N	29.03	N	N	N	N	Pos	Yes
PY21-118	14.28	14.77	16.65	16.96	bleed	N	N	N	N	Pos	Yes
PY21-119	30.80	19.37	33.38	N	N	N	N	N	N	Pos	Poor Quality
PY21-120	20.82	19.86	23.51	N	N	N	N	N	N	Pos	Yes
PY21-121	31.77	21.84	38.68	N	N	N	N	N	N	Pos	NA
PY21-122	20.30	19.79	N	N	24.19	22.83	N	N	N	Pos	Yes
PY21-123	33.87	20.09	N	N	35.94	35.00	N	N	N	Pos	NA
PY21-124	15.97	16.11	19.03	N	19.46	N	N	N	N	Pos	Yes
PY21-125	15.70	16.22	N	N	19.82	18.24	N	N	N	Pos	Yes
PY21-126	16.06	18.75	18.96	N	19.39	N	N	N	N	Pos	Yes
PY21-127	22.22	20.64	N	N	26.02	24.65	N	N	N	Pos	Yes
PY21-128	21.98	20.87	24.83	N	N	N	N	N	N	Pos	Yes
PY21-129	20.17	19.03	N	N	24.15	22.76	N	N	N	Pos	Yes
PY21-130	13.51	13.95	N	N	17.01	15.45	N	N	N	Pos	Yes
PY21-131	21.28	19.21	N	N	24.90	23.46	N	N	N	Pos	Yes
PY21-132	17.65	17.93	N	N	21.17	19.65	N	N	N	Pos	Yes
PY21-133	20.26	18.39	N	N	23.69	22.23	N	N	N	Pos	Yes
PY21-134	25.54	21.90	N	N	28.63	27.25	N	N	N	Pos	Yes
PY21-135	14.41	15.68	N	N	18.10	16.52	N	N	N	Pos	Yes
PY21-136	25.85	25.41	28.61	N	N	N	N	N	N	Pos	Yes
PY21-137	31.81	19.09	35.50	N	N	N	N	N	N	Pos	NA
PY21-138	23.55	23.28	26.85	N	N	N	N	N	N	Pos	Yes
PY21-139	27.80	25.90	32.56	N	N	N	N	N	N	Pos	Yes
PY21-140	22.14	19.18	25.92	N	N	N	N	N	N	Pos	Yes
PY21-141	24.45	21.17	27.55	N	N	N	N	N	N	Pos	Yes
PY21-142	21.66	19.60	25.67	N	N	N	N	N	N	Pos	Yes
PY21-143	19.74	19.96	23.56	N	N	N	N	N	N	Pos	Yes
PY21-144	18.41	19.73	22.74	N	N	N	N	N	N	Pos	Yes
PY21-145	19.69	19.52	23.36	N	N	N	N	N	N	Pos	Yes
PY21-146	23.05	18.79	26.89	N	N	N	N	N	N	Pos	Yes
PY21-147	19.34	20.56	22.75	N	N	N	N	N	N	Pos	Yes
PY21-148	15.89	17.79	19.03	N	N	N	N	N	N	Pos	Yes
PY21-149	15.73	17.85	18.83	N	N	N	N	N	N	Pos	Yes
PY21-150	20.13	19.64	23.67	N	N	N	N	N	N	Pos	Yes

PY21-151	22.19	20.42	26.52	N	27.03	N	N	N	N	Pos	Yes
PY21-152	23.81	21.47	27.47	N	N	N	N	N	N	Pos	Yes
PY21-153	21.22	22.38	24.78	N	N	N	N	N	N	Pos	Yes
PY21-154	21.21	22.96	25.05	N	N	N	N	N	N	Pos	Yes
PY21-155	26.80	22.71	29.56	N	N	N	N	N	N	Pos	Yes
PY21-156	19.66	21.11	23.17	N	N	22.10	N	N	N	Pos	Yes
PY21-157	15.24	17.24	18.52	N	19.17	N	N	N	N	Pos	Yes
PY21-158	12.73	15.10	N	N	17.01	15.36	N	N	N	Pos	Yes
PY21-159	28.52	22.35	N	N	33.10	31.57	N	N	N	Pos	Yes
PY21-160	15.45	17.75	N	N	19.02	17.32	N	N	N	Pos	Yes
PY21-161	16.35	17.78	N	N	20.72	19.01	N	N	N	Pos	Yes
PY21-162	27.73	25.54	N	N	33.53	32.05	N	N	N	Pos	Yes
PY21-163	20.61	21.03	N	N	25.44	23.79	N	N	N	Pos	Yes
PY21-164	20.16	22.00	N	N	24.11	22.50	N	N	N	Pos	Yes
PY21-165	16.48	18.52	N	N	20.18	18.54	N	N	N	Pos	Yes
PY21-166	17.52	19.45	19.99	N	20.62	N	N	N	N	Pos	Yes
PY21-167	16.47	18.72	N	N	20.13	18.51	N	N	N	Pos	Yes
PY21-168	14.16	19.68	N	N	17.71	15.99	N	N	N	Pos	Yes
PY21-169	18.30	20.40	N	N	21.70	20.26	N	N	N	Pos	Yes
PY21-170	15.00	17.13	N	N	18.88	17.29	N	N	N	Pos	Yes
PY21-171	15.83	18.01	N	N	19.38	17.77	N	N	N	Pos	Yes
PY21-172	23.04	20.52	N	N	27.66	26.22	N	N	N	Pos	Yes
PY21-173	16.49	18.65	N	N	20.39	18.83	N	N	N	Pos	Yes
PY21-174	17.35	19.66	N	N	20.92	19.42	N	N	N	Pos	Yes
PY21-175	17.15	18.86	N	N	21.45	19.88	N	N	N	Pos	Yes
PY21-176	14.79	17.10	N	N	18.51	16.93	N	N	N	Pos	Yes
PY21-177	19.42	20.59	N	N	23.24	21.71	N	N	N	Pos	Yes
PY21-178	16.11	18.31	N	N	19.60	18.03	N	N	N	Pos	Yes
PY21-179	17.56	19.73	20.11	20.63	bleed	N	N	N	N	Pos	Yes
PY21-180	18.64	20.81	N	N	22.28	20.66	N	N	N	Pos	Yes
PY21-181	15.48	17.43	N	N	19.82	18.22	N	N	N	Pos	Yes
PY21-182	20.09	22.15	N	N	24.82	23.23	N	N	N	Pos	Yes
PY21-183	19.26	18.21	N	N	23.04	21.45	N	N	N	Pos	Yes
PY21-184	16.12	17.41	N	N	20.51	18.92	N	N	N	Pos	Yes
PY21-185	14.35	16.70	N	N	18.43	16.77	N	N	N	Pos	Yes
PY21-186	14.93	17.23	N	N	18.88	17.22	N	N	N	Pos	Yes
PY21-187	12.42	17.55	N	N	16.44	14.86	N	N	N	Pos	Yes
PY21-188	21.31	19.21	23.88	24.33	bleed	N	N	N	N	Pos	Yes
PY21-189	21.52	20.86	N	N	26.20	24.90	N	N	N	Pos	Yes
PY21-190	16.63	18.69	N	N	20.40	18.97	N	N	N	Pos	Yes
PY21-191	23.43	22.34	N	N	27.54	26.20	N	N	N	Pos	Yes
PY21-192	23.58	23.04	N	N	27.43	26.15	N	N	N	Pos	Yes
PY21-193	16.16	18.10	N	N	19.76	18.33	N	N	N	Pos	Yes
PY21-194	24.84	19.53	N	N	28.52	27.10	N	N	N	Pos	Yes
PY21-195	19.14	20.79	N	N	22.81	21.42	N	N	N	Pos	Yes
PY21-196	22.72	21.56	25.61	26.09	bleed	N	N	N	N	Pos	Poor Quality
PY21-197	16.83	18.73	N	N	20.50	19.07	N	N	N	Pos	Yes
PY21-198	18.09	19.58	N	N	22.76	21.43	N	N	N	Pos	Yes
PY21-199	18.31	20.23	N	N	21.88	20.52	N	N	N	Pos	Yes
PY21-200	17.47	19.48	N	N	20.77	19.39	N	N	N	Pos	Yes
PY21-201	32.62	23.67	N	N	N	N	N	N	N	Neg	NA

Abbreviations: bleed, signifies low signal from orange (452R) detected in red (484K); N, no positive signal detected; N2, SARS-CoV-2 N2 target; NA, no amplicon sequence generated; Neg, negative; Pos, positive; SNP, single nucleotide polymorphism detected in the Spike SNP rRT-PCR; "-", altered RNase P curve from high N2 signal
